# Vulvar squamous intraepithelial neoplasia epithelial thickness in hairy and non-hairy sites: a single center experience from China

**DOI:** 10.3389/fonc.2023.1254820

**Published:** 2023-10-03

**Authors:** Jingjing Xiao, Ziren Chen, Yinping Xiao, Long Sui, Chao Wang, Qing Cong

**Affiliations:** ^1^ Obstetrics and Gynecology Hospital of Fudan University, Shanghai, China; ^2^ Shanghai Key Laboratory of Female Reproductive Endocrine Related Diseases, Obstetrics and Gynecology Hospital of Fudan University, Shanghai, China; ^3^ Department of Pathology, Obstetrics and Gynecology Hospital of Fudan University, Shanghai, China; ^4^ Department of Cervical Disease Center, Obstetrics and Gynecology Hospital of Fudan University, Shanghai, China

**Keywords:** vulvar intraepithelial neoplasia, vulva, HPV, squamous intraepithelial lesion, treatment, thickness

## Abstract

**Introduction:**

A large-sample study focusing on VIN lesions of a more precise thickness is needed to help guide clinical treatment. This study aimed to investigate the depth of vulvar intraepithelial neoplasia (VIN) and involved skin appendages to provide evidence for laser surgery.

**Methods:**

The study retrospectively enrolled and analyzed the clinical characteristics of VIN patients in the obstetrics and gynecology department of a university hospital between January 1, 2019 and December 30, 2021. The study further explored the thickness of epithelium and skin appendages of 285 women with low-grade VIN (VIN1) and 285 women with high-grade VIN (VIN2/3).

**Results:**

The study included 1,139 (80%) VIN1 and 335 (20%) VIN2/3 cases. The VIN1 and VIN2/3 groups showed a significant difference in human papillomavirus infection (P<0.01) but not in cytology (P = 0.499). Most (89.90%, 1,325) cases occurred in one area of the vulva, whereas 10.11% were multifocal. VIN commonly occurred on the posterior fourchette (76.85%), labia majora (11.61%), and labia minora (9.92%). The VIN2/3 group reported a significantly higher positive rate for concurrent cervical and vaginal intraepithelial neoplasia (160 of 285) than the VIN1 group (321 of 953) (P=0.000). The involved epithelial thicknesses in VIN2/3 and VIN1 were 0.69 ± 0.44 and 0.49 ± 0.23 mm, respectively, both of which were greater than the corresponding noninvolved epithelial thickness (0.31 ± 0.19 and 0.32 ± 0.10 mm, P<0.001 and P<0.001, respectively). In cases of appendage involvement, the VIN thickness was 1.98 ± 0.64 mm.

**Conclusions:**

VIN thickness was generally ≤1 mm for the superficial lesions in non-hairy areas. However, for lesions extending onto hairy areas, the thickness was approximately 3 mm, leading to the destruction of involved skin appendages.

## 1 Introduction

In 2015, the International Society for the Study of Vulvovaginal Disease proposed a revised classification of vulvar intraepithelial neoplasia (VIN) terminology. This classification included subtypes such as low-grade squamous intraepithelial lesion (LSIL) and high-grade squamous intraepithelial lesion (HSIL), alongside the VIN differentiated type (1). LSIL and HSIL correspond to the former VIN1 and VIN2/3 nomenclature, respectively. VIN2/3 is more prevalent in younger women (2) and is considered a premalignant condition. This condition is related to invasive vulvar squamous cell carcinomas (VSCC), which account for over 80% of vulvar malignancies.

The management of VIN remains challenging owing to the lack of a clear consensus regarding the best treatment modality. VIN therapy must be individualized; therefore, comparing therapies to determine the optimal treatment is often difficult. Patients with VIN1 are recommended to undergo observation without treatment owing to the high rate of spontaneous regression. For patients with visible VIN1 lesions or those whose VIN1 lesions do not improve during observation, drugs, physical therapy, and surgical procedures can be considered. Current treatment options for VIN2/3 include local surgical excision (consisting of the removal of all visible lesions using a scalpel and electrosurgery), chemotherapy (including cidofovir, photodynamic therapy, and imiquimod), photodynamic therapy, laser ablation, and vaccination (3). Local surgical excision, often in the form of vulvectomy, can be disfiguring, emotionally distressing, and cause sexual problems in many women. Additionally, the incidence of VIN among younger women has been increasing, prompting the consideration of conservative therapy as a viable option. Alternative conservative topical chemotherapy, utilizing immune-modulating agents, and antiviral therapy have varying disadvantages. These include high rates of ulceration and unclear success response rates, ranging 26–70% (4–6). Photodynamic therapy has demonstrated numerous limitations, including a high rate of treatment failure, immunosuppressive effects, and consequent increases in direct and non-direct costs (7). Laser surgery, which uses a high-energy light beam, has been proposed as a surgical intervention for various cases of VIN. This approach has yielded mixed success, with reports indicating generally favorable tolerance, satisfactory healing, and minimal sexual dysfunction (8, 9).

However, the risk of residual disease or VIN recurrence exists in different treatment methods owing to unclear identification of the lesion’s macroscopic characteristics. Notably, different areas of the vulva exhibit variations in skin structure. Few studies have investigated the depth of epithelial and involved appendages and yielded consistent results. These studies suggested that depths of 1.0 mm and 2.0–2.5 mm in non-hairy and hairy sites, respectively, were appropriate for successful treatment (10, 11). However, these studies included only a small number of patients. Thus, a study with a large sample size focusing on VIN lesions with a more precise thickness is required to help guide clinical treatment. In this study, we aimed to describe the depth of involved and noninvolved vulvar epithelium and appendages in women with VIN and recommend the optimal depth for epithelial ablation during laser surgery.

## 2 Materials and methods

### 2.1 Patients

This was a single-center retrospective study conducted in a large obstetrics and gynecology hospital in China. We enrolled patients who underwent colposcopy-directed biopsy or vulvar surgical vulvectomy and subsequently diagnosed with VIN1 and VIN2/3 between January 1, 2019 and December 30, 2021. Patients with an incomplete medical history, VIN with warts (condyloma acuminata), and who were lost to follow-up were excluded. Approval was obtained from the relevant institutional review board before data extraction was commenced, and all women provided consent to participate in the study. Finally, 285 patients with VIN2/3 were enrolled in the study. Considering the large number of VIN1 cases, we randomly selected 285 VIN 1 patients who were diagnosed during the study recruitment period.

### 2.2 Cytology and human papillomavirus testing

Cervical or vaginal cytology tests were interpreted and reported by two pathologists based on the 2014 Bethesda System. Human papillomavirus (HPV) testing was performed using a fluorescence-based multiplex real-time HPV DNA genotyping kit (Bioperfectus, Jiangsu, China) capable of detecting both high-(16, 18, 26, 31, 33, 35, 39, 45, 51, 52, 53, 56, 58, 59, 66, 68, 73, and 82) and low-risk HPV types (6,11, and 81).

### 2.3 Histological technique and anatomicopathological features

We examined tissue specimens from all study participants Specimens were collected through biopsy or local surgical vulvectomy, subsequently fixed in buffered formalin, and embedded in paraffin. We stained 4-μm sections of paraffin-processed samples with hematoxylin and eosin. Two experienced pathologists scanned and reviewed all digital slides. All margin diagnoses were negative for intraepithelial lesions or invasive cancer. To compare the vulvar epithelium thickness before and after formalin fixation, seven radical vulvectomy samples classified as International Federation of Gynecology and Obstetrics phase I VSCC were selected. Each sample included a frozen section diagnosis of the vulvar margin, as well as its corresponding formalin-fixed and paraffin-embedded (FFPE) sample. All margin diagnoses were negative for intraepithelial lesions or invasive cancer. In total, we retrieved 21 paired sections, 3 from each case. Epithelium thickness was measured on whole-slide images, targeting similar sites on the paired sections.

Surface keratinization or surface separated from the epidermis rendered the use of surface as a reference point inaccurate. Consequently, we initiated vertical measurements at the stratum corneum-granulosum junction and extended them to the basal layer. We measured multiple foci and recorded the maximum values. We also obtained the thickness of involved and noninvolved epithelium or appendages in the same section. All available data were recorded, including patient cytology records, history of HPV and CIN/VaIN disease (excluding other diseases, such as diabetes, hypertension, and autoimmune diseases), and age.

### 2.4 Statistical analysis

Statistical analyses were performed using SPSS Statistics for Windows, version 16.0 (SPSS Inc., Chicago, IL, USA). We utilized independent samples t-tests and chi-squared tests to assess differences between groups. Statistical significance was set at P<0.05.

## 3 Results

A total of 1,474 women who underwent vulvar biopsy or local surgical vulvectomy between January 1, 2019 and December 30, 2021 were diagnosed with VIN (**Table 1**), with an average of 42.72 ± 14.31 years. Of them, 1,139 (80%) and 335 (20%) were diagnosed with VIN1 and VIN2/3, respectively. In addition, women in the VIN2/3 group were significantly older than those in the VIN1 group (P<0.01). We also found a significant difference in HPV infection rate (P<0.01) but not in cytology (P = 0.499) between the VIN1 and VIN2/3 groups. For the VIN lesions, 90% (1,325 of 1,474) were unifocal, and 10% (149) were multifocal. In our study, VIN was commonly found on the posterior fourchette (76.85%), labia majora (11.61%), and labia minora (9.92%). We recorded 1,374 of 1,678 (77.83%) VIN lesions in non-hairy areas and 372 (22.17%) in hairy areas. Cervical squamous intraepithelial neoplasia (CIN) and/or vaginal squamous intraepithelial neoplasia (VaIN) were observed in 38.85% (481 of 1,238) of VIN cases without a history of CIN/VaIN/any other disease. We noted a significantly higher positive rate for concurrent CIN and VaIN in the VIN2/3 group (56.14%, 160 of 285) compared with that in the VIN1 group (33.68%, 321 of 953) (P=0.000).

**Table 1:** Clinical Characteristics of the 1474 Women with Vulvar Intraepithelial Neoplasia.

	Total	VIN2/3		VIN1		P
	n=1474	n=335		n=1139		
**Age(y)**	42.72±14.31	44.69±14.75		41.28±13.82		0.007
**Cytology**						0.499
≤LSIL	1279	287	85.67%	992	87.09%	
≥HSIL	195	48	14.33%	147	12.91%	
**HPV infection**						0.008
Yes	1357	320	95.52%	1037	91.04%	
NO	117	15	4.48%	102	8.96%	
**Number of lesion site**						<0.001
1	1325	301	89.85%	1024	89.90%	
≥2	149	34	10.15%	115	10.10%	
**Lesion site**						<0.001
**non-hairy**						
Posterior forchette	1036	182	46.79%	854	66.25%	
labia minora	182	58	14.91%	124	9.62%	
Navicular fossa	52	14	3.60%	38	2.95%	
Urethral opening	29	13	3.34%	16	1.24%	
clitoris	7	4	1.03%	3	0.23%	
**hairy**						
labia majora	240	80	20.57%	160	12.41%	
interlabial grooves	68	9	2.31%	59	4.58%	
perianal areas	64	29	7.46%	35	2.72%	
Accompanied with cervical/vaginal SIL*						0.002
Yes	481	160	56.14%	321	33.68%	
NO	757	125	43.86%	632	66.32%	
**History with CIN/VaIN/any other disease**						0.538
Yes	236	50	14.93%	186	16.33%	
NO	1238	285	85.07%	953	83.67%	

HSIL, high-grade squamous intraepithelial lesion; LSIL, low-grade squamous intraepithelial lesion; ≤LSIL, atypical squamous cells of undetermined significance, no intraepithelial or malignant lesions, or low-grade squamous intraepithelial lesion; SIL, squamous intraepithelial lesion; *VIN cases were accompanied with cervical/vaginal SIL and they had no history of any other disease at the same time.

We randomly selected 285 VIN1 patients who were diagnosed during the same period as VIN-2/3 patients, all of whom had no history of CIN/VaIN. The clinical characteristics of the women with VIN1 and VIN2/3 are shown in **Supplementary 1**. In the VIN1 group, six cases had two lesion sites, specifically the posterior fourchette and labia majora. In the VIN2/3 group, 20 cases had 2 lesion sites, and 3 cases had 3 lesion sites, specifically the posterior fourchette, labia majora, and perianal areas. In the VIN1 group, CIN/VaIN 1 and CIN/VaIN 2/3 were detected in 35 (12.28%) and 8 (2.81%) cases, respectively. Two patients (0.70%) also had squamous cell carcinoma of the cervix (SCC). In the VIN2/3 group, CIN/VaIN 1, CIN/VaIN 2/3, SCC, and vaginal squamous carcinoma (VaSCC) were detected in 90 (31.6%), 59 (20.7%), 8 (2.8%), and 3 (1.1%) patients, respectively.

**Table 2**, **Figures 1**, **2** show the epithelial thickness of VINs in different sites. We examined 291 and 309 sections of tissue from 285 patients with VIN1 and 285 patients with VIN 2/3, respectively. Of the 600 tissue sections, VIN was detected on the posterior fourchette (45.33%), labia majora (18.83%), and labia minora (14.83%). The maximum depths of epithelial lesions were 1.6 mm and 2.75 mm in the VIN1 and VIN 2/3 groups, respectively. In the VIN2/3 group, significant differences in the thickness of involved and noninvolved epithelia were detected across all vulvar sites (P<0.05). Moreover, in the VIN1 group, the thickness of involved epithelia was greater than that of the noninvolved epithelia, except in the clitoris, urethral opening, and navicular fossa. The thickness of involved epithelium were 0.69 ± 0.44 mm and 0.49 ± 0.23 mm in the VIN2/3 and VIN1 groups, respectively (P=0.000). However, the depth of noninvolved epithelia was projected to be consistent across all VIN grades. We found that 32.81% (187 of 570) of VINs were involved in hairy areas. The rates of involvement in hairy areas were 28.07% (80 of 285) and 37.54% (107 of 285) in the VIN1 and VIN2/3 groups, respectively. The most common lesion site in non-hairy areas was the posterior fourchette in both groups. Conversely, the labia majora was the most common lesion site in hairy areas. We noted significant differences in the epithelial thickness between VINs in non-hairy and hairy areas (0.52 ± 0.30 mm vs. 0.78 ± 0.45 mm, P<0.001).

**Table 2:** Involved and noninvolved vulvar epithelial thickness in patients of different sites.

Site		VIN2/3						VIN1					
	total	No. patients	Involved	(range)	Noninvolved	(range)	P	No. patients	Involved	(range)	Noninvolved	(range)	P
**Non-hariy**	413	202	0.59±0.36	0.15-2.37	0.30±0.20	0.08-1.20	<0.001	211	0.46±0.31	0.15-1.40	0.31±0.10	0.18-0.64	<0.001
Posterior forchette	272	153	0.62±0.39	0.15-2.37	0.30±0.21	0.08-1.2	<0.001	119	0.50±0.24	0.29-1.40	0.33±0.11	0.22-0.64	<0.001
labia minora	89	26	0.47±0.22	0.25-1.19	0.30±0.20	0.10-0.70	0.006	63	0.42±0.16	0.15-0.80	0.26±0.05	0.18-0.37	<0.001
clitoris	14	5	0.59±0.05	0.51-0.65	0.31±0.09	0.23-0.47	<0.001	9	0.43±0.14	0.28-0.65	0.36±0.10	0.26-0.51	0.224
Navicular fossa	19	10	0.54±0.31	0.36-1.40	0.27±0.07	0.11-0.47	0.023	9	0.30±0.10	0.15-0.43	0.23±0.03	0.19-0.28	0.059
Urethral opening	19	8	0.52±0.13	0.31-0.7	0.27±0.13	0.11-0.50	0.002	11	0.48±0.19	0.28-0.82	0.36±0.10	0.26-0.51	0.072
**Hariy**	187	107	0.92±0.51	0.1-2.75	0.33±0.18	0.12-1.18	<0.001	80	0.58±0.27	0.24-1.60	0.36±0.11	0.21-0.78	<0.001
interlabial grooves	35	10	0.66±0.25	0.40-1.10	0.30±0.11	0.15-0.47	0.001	25	0.52±0.21	0.30-1.00	0.31±0.07	0.21-0.45	<0.001
labia majora	113	70	0.95±0.52	0.25-2.75	0.30±0.19	0.12-1.18	<0.001	43	0.63±0.29	0.32-1.6	0.37±0.11	0.27-0.64	<0.001
perianal areas	39	27	0.93±0.52	0.10-1.82	0.40±0.16	0.18-0.91	<0.001	12	0.57±0.27	0.24-1.15	0.41±0.14	0.28-0.78	0.048
**Total**	600	309	0.69±0.44	0.10-2.75	0.31±0.19	0.08-1.2	<0.001	291	0.49±0.23	0.15-1.60	0.32±0.10	0.18-0.78	<0.001

VIN, Vulvar Intraepithelial Neoplasia.

**Figure 1:**
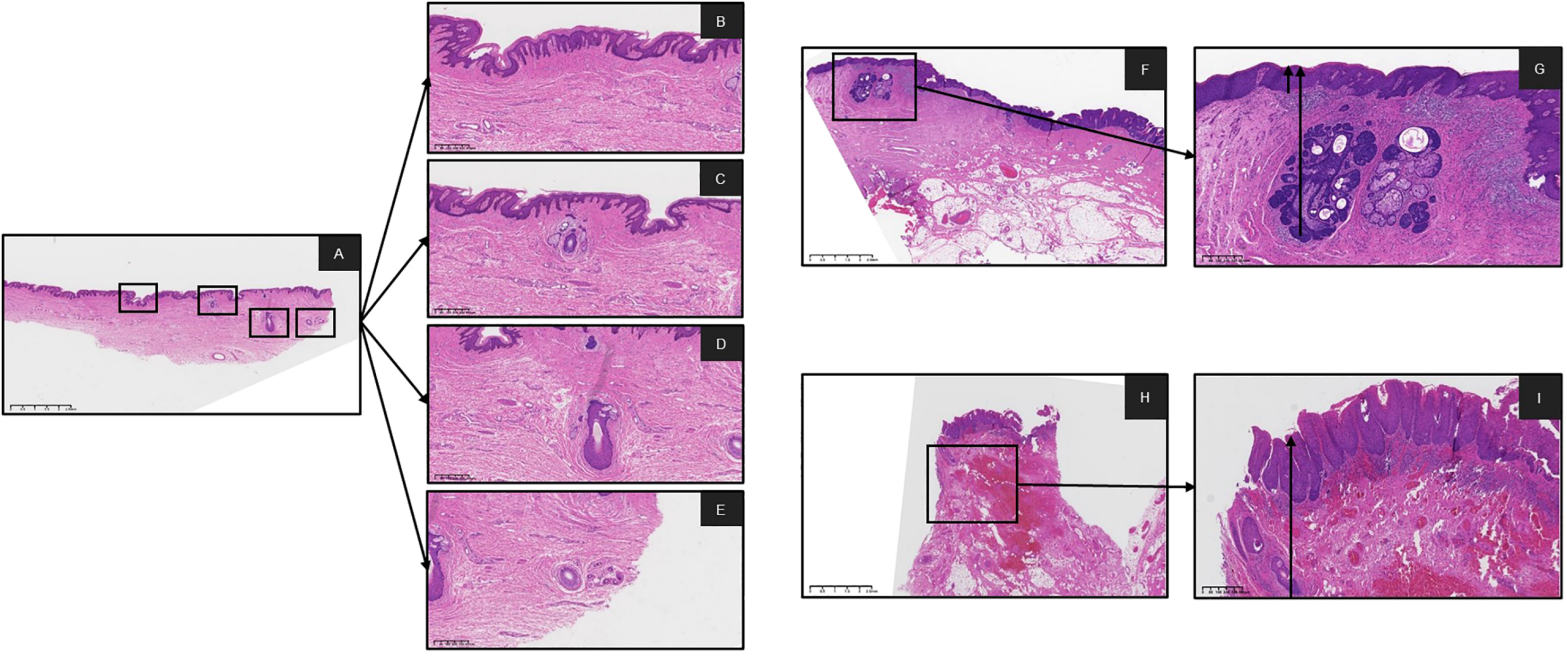
Digital pathology slides were scanned by K-scanner (KF-BIO-120, digital pathology slides scanner, KFBIO) and reviewed on K-viewer software. **(A)** was showing normal issue of epithelial and skin appendages on the same slide HE X 1. **(B-E)** were showing the epithelial, Hair Follicles, Sebaceous Gland and Sweat Gland, on the A slide HE X 4, respectively Measurement of depth from the basal layer to the surface of the squamous epithelium was obtained as the arrow was pulled at the locus of normal tissue. **(F, G)** with the involved epithelium and Sebaceous Gland on the slide HE X 1, HE X 4, respectively. **(H, I)** with the involved epithelium and Hair Follicles on the slide HE X 1, HE X 4, respectively. (HE, hematoxylin-eosin staining).

**Figure 2:**
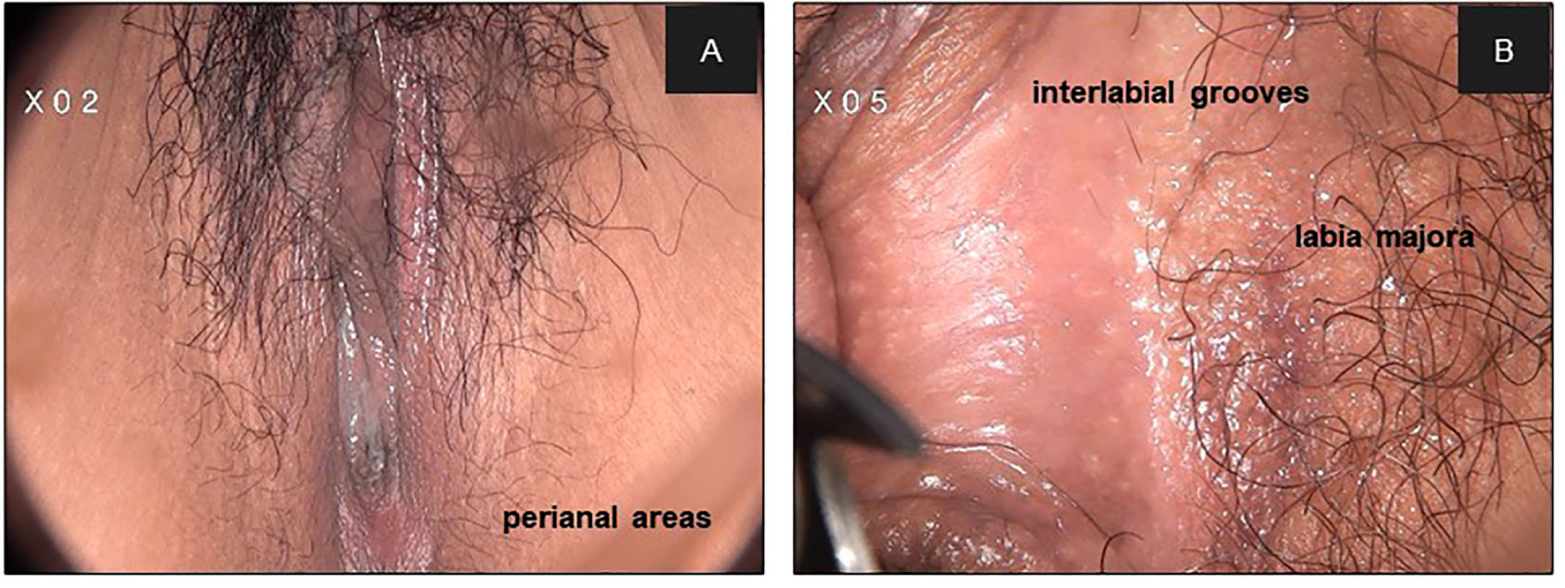
The hairy parts of the vulva were showed under colposcopy. **(A) **2X; **(B) **5X.

**Figure 3:**
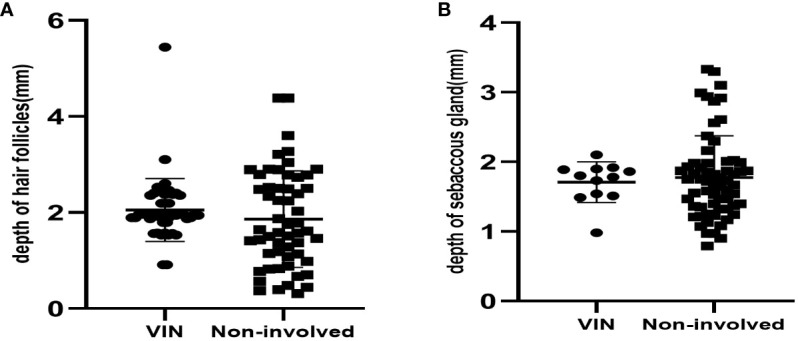
**(A)** The depth of hair follicles in 45 VINs and 56 non-involved cases. **(B)** The depth of sebaceous gland in 12VINs and 63 non-involved cases.

**Table 3** shows the depth of involved epithelial and skin appendages in VIN and noninvolved tissue. Compared with nondysplastic samples, we found no significant difference in the depth of stratum corneum between the VIN groups. The thickness of involved skin appendages in VIN ranged 0.91–5.44 mm (mean depth, 1.98 ± 0.64 mm), whereas that of noninvolved skin appendages ranged 0.26–4.38 mm (mean depth, 1.66 ± 0.85 mm). VIN appeared to affect hair follicles in only one patient, with the depth reaching 5.44 mm. The thickness of epithelium of the involved skin appendages in VIN was consistently greater than that of the involved epithelium at the same section (1.98 ± 0.61 mm vs. 1.01 ± 0.52 mm, P<0.001). Hair follicles represented the most commonly involved appendage, followed by sebaceous glands. The involvement of sweat glands was not detected in any VINs (**Figure 3**).

**Table 3:** Depth of epithelial and skin appendages in vulvar intraepithelial neoplasia and noninvolved tissue.

	VIN			Non-involved			P
Diagnosis	No. patients	Mean±SD(mm)	Range(mm)	No. patients	Mean±SD(mm)	Range(mm)	
**Stratum Corneum**	57	0.10±0.06	0.04-0.25	47	0.08±0.08	0.01-0.52	0.331
**Epidermis**	57	1.01±0.52	0.25-2.75	57	0.28±0.16	0.12-1.08	<0.001
**Appendage**	57	1.98±0.61	0.91-5.44	186	1.66±0.85	0.26-4.38	/
Hair Follicles	45	2.05±0.66	0.91-5.44	56	1.86±0.99	0.31-4.38	0.271
Sebaceous Gland	12	1.71±0.29	0.98-2.1	63	1.77±0.60	0.79-3.33	0.715
Sweat Gland	/	/	/	67	1.40±0.87	0.26-4.12	/

As shown in **Supplementary 2**, the thickness of involved epithelia in all VIN grades was consistently greater than that of the noninvolved epithelia across all age groups. We observed a significant decrease of thickness with age in both noninvolved and involved epithelia in all VIN grades (P<0.001 for all comparisons). In comparisons between the VIN2/3 and VIN1 groups, the differences in thickness of the involved epithelia were statistically significant across the different age groups, including pre- and postmenopausal women (P<0.001 for all comparisons). Comparisons of the thickness of noninvolved epithelia showed no significant differences between the VIN2/3 and VIN1 groups in subgroup-level (age, premenopausal and postmenopausal group; P>0.05 for all comparisons).

To compare the vulvar epithelium thickness before and after FFPE treatment, we analyzed 21 pairs of frozen and corresponding FFPE-treated sections. The epithelial thickness was 0.32 ± 0.18 mm and 0.31 ± 0.11 mm for the frozen and FFPE sections, respectively, indicating no significant difference in size changes due to tissue fixation (P=0.56).

## 4 Discussion

This study aimed to investigate the depth of involved and noninvolved vulvar epithelium and appendages in women with VIN to provide evidence for laser surgery. Our study showed that the value of cytology results was limited in identifying the severity of VIN lesions as no differences was observed between cytology results of the VIN1 and VIN2/3 groups. However, when the cytology results are positive for HSIL, care should be taken to avoid missing serious lesions during colposcopy. In our study, most VINs were associated with HPV infection, consistent with other studies that reported HPV positivity rates >80% (12–15), confirming the cause-and-effect relation.

VINs tend to be multifocal and multicentric, with approximately 18–56% VIN patients simultaneously having cervix, vaginal, and anal lesions (2, 16–19). In our study, 31.58% of VIN2/3 cases were concurrent with LSIL, 20.27% with HSIL, 2.81% with SCC, and 1.05% with VaSCC; VIN2/3 cases were more likely to be accompanied by cervical and vaginal lesions. Our data supported the concept that HPV-related disease can manifest as multicentric lesions in the lower female genital tract rather than being confined to one particular organ. Thus, detecting VIN on clinical examination should prompt a thorough examination from the cervix to the perianal area (20). Meanwhile, owing to the lack of effective screening methods for VIN2/3, a comprehensive and careful examination of the vulvar and perianal areas remains the main method for the early detection of VIN2/3.

The thickness of involved epithelium was ≤1 mm in 87.67% (526 of 600) of VIN cases. However, 61 (of 301, 20.37%) VIN2/3 and 13 (of 291, 4.47%) VIN1 cases showed an epithelial thickness >1 mm. Further, the number of VIN2/3 lesion sites with depths >1 mm in hairy sites (including the labia majora and perianal areas) was higher (at 49 of 97 cases). Meanwhile, 53.85% (7 of 13) VIN1 cases occurred in hairy sites (including the labia majora and perianal areas). Therefore, a treatment depth of 1 mm may be sufficient for most VIN cases, although patients with lesions in hairy areas should be monitored. According to our results, more than half of the patients with lesions in hairy sites had lesion depths of 1–3 mm.

In our results, lesions in hairy sites were thicker than those in non-hairy areas. Histologically, non-hairy sites include the clitoris, labia minora, and posterior fourchette, which are characterized by the absence of hair follicles and sweat glands. Hair-bearing skin of the inter-labial grooves, labia majora, lateral perineal, and perianal areas consist of skin appendages, including hair follicles, sebaceous glands, and apocrine and exocrine sweat glands. The upper parts of hair root sheath and lining of the sebaceous gland duct are susceptible to the extension of epithelial lesions owing to their contiguity with the surface epithelium and the presence of similar cell types. Exocrine and apocrine gland ducts are lined by their own independent epithelium, which are not contiguous with the surface epithelium. Involvement of hair root sheaths to depths of 0.8–2.5 mm has been documented (21). Involvement of the sebaceous duct occurs less often, secondary to that of the sheath.

Baggish and Dorsey suggested a uniform depth of 3 mm for laser vaporization for all areas of the vulva (22). Buckley et al. conducted a study involving 28 patients with involved skin appendages and reported that CO2 laser eradication of the skin to a depth of 5 mm can eliminate all atypical epithelium in skin appendages. However, it is unclear whether tissues within the 5–10 mm depth should also be destroyed to ensure that no appendages remain, considering that the appendages may penetrate deeper than 5 mm (21). Based on our results, the depth of lesions extending into the appendages was much deeper than that of the involved epithelium in the same section. We found that superficial lesions in non-hairy areas were vaporized by the laser to a ≤1 mm depth; however, lesions extending onto hairy areas were vaporized to 3 mm to destroy involved skin appendages. Our results were similar to previous findings of a study involving only 29 patients, of which only 5 cases had involved skin appendages (23). However, in our study, we detected only one case with involved appendages with a lesion depth >5 mm. Therefore, we were unable to provide evidence supporting laser surgery with a >5 mm depth for VIN patients.

VIN treatment aims to completely destroy the lesion, improve symptoms, exclude invasion, preserve normal vulvar anatomy and function, and avoid recurrence (3). VIN has a recurrence rate of 20–36.7% despite treatment (24, 25), with 2–15% of cases progressing to vulvar cancer (26, 27). The risk factors for recurrence and progression of VIN remain poorly understood. To eliminate VIN and avoid recurrence, health-care professionals must understand the structure of the skin and recognize the involvement of appendages.

In summary, the epithelium of VIN2/3 lesions was thicker than that of VIN1 lesions, especially in hairy areas. The depth of involvement of appendages was greater than the thickness of epithelial lesion in the same section. The lesion depth in hairy areas was 1–3 mm, with or without appendage involvement. This was deeper than the lesion depth in non-hairy areas, which was approximately 1 mm. Hence, the removal of involved epithelium and appendages would be advisable for laser surgery.

## Data availability statement

The original contributions presented in the study are included in the article/**Supplementary Material**. Further inquiries can be directed to the corresponding authors.

## Ethics statement

The studies involving humans were approved by Fudan University Obstetrics and Gynecology Hospital Ethical committee approval. The studies were conducted in accordance with the local legislation and institutional requirements. The participants provided their written informed consent to participate in this study. Written informed consent was obtained from the individual(s) for the publication of any potentially identifiable images or data included in this article.

## Author contributions

JX: Writing – original draft, Writing – review & editing, Formal Analysis, Investigation, Methodology. ZC: Methodology, Writing – original draft. YX: Formal Analysis, Writing – original draft. LS: Conceptualization, Formal Analysis, Methodology, Writing – original draft, Writing – review & editing, Investigation. CW: Conceptualization, Formal Analysis, Writing – original draft, Writing – review & editing. QC: Conceptualization, Formal Analysis, Methodology, Writing – original draft, Writing – review & editing, Data curation, Investigation.
