# Supplementary material for: Vulvar squamous intraepithelial neoplasia epithelial thickness in hairy and non-hairy sites: a single center experience from China
Source: Front Oncol. 2023 Oct 3;13:1254820. doi: 10.3389/fonc.2023.1254820 (PMC10579793; doi:10.3389/fonc.2023.1254820)
Supplement: Supplementary file 1 [file Table_1.docx]

| **Supplementary 1. Clinical Characteristics of the 570 Women With Vulvar Intraepithelial Neoplasia** | | | | | | |  |
| --- | --- | --- | --- | --- | --- | --- | --- |
|  | **Total** | **VIN2/3** | | **VIN1** | | **P** |  |
|  | **n=570** | **n=285** | | **n=285** | |  |  |
| **Age(y)** |  | 44.53±14.75 | | 44.45±14.59 | | 0.958 |  |
| **Cytology** |  |  |  |  |  | 0.010 |  |
| ≤LSIL | 523 | 253 | 88.77% | 270 | 94.74% |  |  |
| ≥HSIL | 47 | 32 | 11.23% | 15 | 5.26% |  |  |
| **HPV infection** |  |  |  |  |  | ＜0.001 |  |
| Yes | 537 | 280 | 98.25% | 257 | 90.18% |  |  |
| NO | 33 | 5 | 1.75% | 28 | 9.82% |  |  |
| **Number of lesion site** |  |  |  |  |  | 0.002 |  |
| 1 | 542 | 263 | 92.28% | 279 | 97.89% |  |  |
| ≥2 | 28 | 22 | 7.72% | 6 | 2.11% |  |  |
| **Appendages Involved** |  |  |  |  |  | ＜0.001 |  |
| **YES** | 57 | 54 | 18.95% | 3 | 1.05% |  |  |
| labia majora | 50 | 47 | 16.49% | 3 | 1.05% |  |  |
| perianal areas | 7 | 7 | 2.46% | 0 | 0.00% |  |  |
| **NO** | 513 | 231 | 81.05% | 282 | 98.95% |  |  |
| **Accompanied with cervical/vaginal SIL** |  |  |  |  |  |  |  |
|  |  |  |  |  |  | ＜0.001 |  |
| **Yes** | 205 | 160 | 56.14% | 45 | 15.79% |  |  |
| LSIL | 125 | 90 | 31.58% | 35 | 12.28% |  |  |
| HSIL | 67 | 59 | 20.70% | 8 | 2.81% |  |  |
| SCC | 10 | 8 | 2.81% | 2 | 0.70% |  |  |
| VSCC | 3 | 3 | 1.05% | 0 | 0.00% |  |  |
| **NO** | 365 | 125 | 43.86% | 240 | 84.21% |  |  |
| HSIL, high-grade squamous intraepithelial lesion; LSIL: low-grade squamous intraepithelial lesion; ≤LSIL: atypical squamous cells of undetermined significance, no intraepithelial or malignant lesions, or low-grade squamous intraepithelial lesion; SIL: squamous intraepithelial lesion. | | | | | | |  |
|  |  |  |  |  |  |  |  |

| **Supplementary 2. Involved and Noninvolved Vulvar Intraepithelial Neoplasia Epithelial Thickness in Patients of Different Ages and Menstrual status** | | | | | | | | | | | | |
| --- | --- | --- | --- | --- | --- | --- | --- | --- | --- | --- | --- | --- |
|  | **VIN2/3** | | | | | | **VIN1** | | | | | |
|  | **No. patients** | **Involved** | **Range** | **Noninvolved** | **Range** | **P** | **No. patients** | **Involved** | **Range** | **Noninvolved** | **Range** | **P** |
| **Age** |  |  |  |  |  |  |  |  |  |  |  |  |
| ≤40 | 145 | 0.75±0.48 | 0.1-2.75 | 0.31±0.18 | 0.08-1.08 | <0.001 | 123 | 0.51±0.27 | 0.22-1.6 | 0.34±0.11 | 0.23-0.78 | <0.001 |
| 41-50 | 48 | 0.71±0.39 | 0.24-1.53 | 0.32±0.23 | 0.1-1.18 | <0.001 | 62 | 0.49±0.20 | 0.3-1.1 | 0.31±0.09 | 0.19-0.56 | <0.001 |
| 51-60 | 55 | 0.60±0.32 | 0.15-1.6 | 0.30±0.20 | 0.1-1.2 | <0.001 | 56 | 0.48±0.20 | 0.15-1.1 | 0.33±0.12 | 0.19-0.64 | <0.001 |
| ≥61 | 61 | 0.58±0.36 | 0.15-2.30 | 0.30±0.18 | 0.1-1.1 | <0.001 | 50 | 0.48±0.21 | 0.15-1.0 | 0.27±0.04 | 0.18-0.4 | <0.001 |
| **Epithelium status** |  |  |  |  |  |  |  |  |  |  |  |  |
| Premenopausal | 216 | 0.74±0.47 | 0.1-2.75 | 0.32±0.21 | 0.08-1.2 | <0.001 | 225 | 0.51±0.24 | 0.15-1.6 | 0.33±0.11 | 0.19-0.78 | <0.001 |
| Postmenopausal | 93 | 0.62±0.36 | 0.15-2.06 | 0.28±0.15 | 0.1-0.8 | <0.001 | 66 | 0.46±0.20 | 0.15-1.1 | 0.28±0.07 | 0.18-0.64 | <0.001 |
